# Novel Iron Parameters in Patients with Type 2 Diabetes Mellitus in Relation to Kidney Function

**DOI:** 10.3390/jcm10163732

**Published:** 2021-08-22

**Authors:** Agnieszka Zapora-Kurel, Łukasz Kuźma, Magdalena Zakrzewska, Marcin Żórawski, Sławomir Dobrzycki, Małgorzata Twardowska-Kawalec, Jolanta Małyszko

**Affiliations:** 1 2nd Department of Nephrology with Hypertension and Dialysis, Medical University of Bialystok, 15-089 Bialystok, Poland; agnieszkazapora2@wp.pl (A.Z.-K.); magdalena.maciorkowska@gmail.com (M.Z.); 2 Department of Invasive Cardiology, Medical University of Bialystok, 15-089 Bialystok, Poland; kuzma.lukasz@gmail.com (L.K.); slawek_dobrzycki@yahoo.com (S.D.); 3 Department of Clinical Medicine, Medical University of Bialystok, 15-089 Bialystok, Poland; mzorawski@wp.pl; 4 Department of Nephrology, Dialysis and Internal Medicine, Medical University of Warsaw, 02-091 Warsaw, Poland; mtwardowskakawalec@gmail.com

**Keywords:** chronic kidney disease, diabetes mellitus, iron metabolism, hepcidin

## Abstract

Background/aims: Anemia of chronic disease is a common feature in diabetes and chronic kidney disease. Hepcidin is the key element involved in iron metabolism; however, studies on new indices of iron status are still ongoing. The aim of the study was to assess novel iron parameters in patients with type 2 diabetes mellitus in relation to kidney function. Methods: The study included 80 type 2 diabetic patients and 23 healthy volunteers. Standard laboratory measurements were used to measure the iron status, complete blood count, creatinine, the estimated glomerular filtration rate (eGFR), serum lipids, and brain natriuretic peptides (BNPs). Commercially available kits were used to measure hepcidin-25, the soluble transferrin receptor (sTfR), growth differentiation factor-15 (GDF-15), and hypoxia-inducible factor-1 alpha. Results: Anemia was present in 65% of the studied patients. The control group was found to have significantly higher hepcidin, sTfR, and GDF-15, and lower hemoglobin and iron. When compared with patients with eGFR values ≥60 mL/min/1.73 m^2^ and <60 mL/min/1.73 m^2^, we found that patients with higher eGFR had higher hemoglobin, ferritin, and HIF-1 alpha, lower BNP, and were younger. We found that levels of HIF-1 alpha are negligible in the studied population and were related to age only in patients with eGFR values ≥60 mL/min/1.73 m^2^. Conclusion: A comprehensive assessment of iron status is rarely performed. Novel biomarkers of iron metabolism are not generally related to kidney function. Whether the assessment of HIF-1 alpha would be a marker of efficient anemia therapy with HIF-prolyl hydroxylase inhibitors is still a matter for further study.

## 1. Introduction

Iron is one of the most common elements on earth and is essential for the proper growth and development of organisms. Imbalance in its quantity can lead to many pathologies. Due to its ability to occur in two forms of oxidation, it participates in the vital process of binding and distributing oxygen, metabolism, cell growth, and proliferation. However, with its ability to accept and transfer electrons, iron can cause severe oxidative stress and tissue damage [1]. Iron homeostasis in the body is maintained by regulators acting at the systemic and cellular levels. The body’s ability to excrete iron is limited; therefore, its amount is particularly controlled at the level of its absorption from the gastrointestinal tract [2]. Hepcidin is the systemic regulator of iron metabolism. It is produced in the liver and depends on the amount of iron in the body [3], the presence of inflammation [4], hypoxia [5,6], and erythropoiesis [7].

Research has disclosed the reciprocal influence between diabetes and iron metabolism. Many of the pathways responsible for iron homeostasis in the body are disrupted in the hyperglycemic environment. On the other hand, excess iron has an effect on insulin action and secretion. Too much iron in the body promotes the development of glucose intolerance, type 2 diabetes [8,9,10,11,12,13,14], and gestational diabetes [15,16]. The mechanisms through which iron contributes to the pathogenesis of diabetes are not yet fully understood, but appear to be multifactorial and may vary depending on the cause of iron overload and its tissue distribution [1]. The relationship between excess iron and the pathogenesis of type 2 diabetes has been observed in patients suffering from haemochromatosis and thalassemia [17,18]. On the other hand, lowering iron concentration may be a factor involved in reducing the risk of developing type 2 diabetes [19]. Phlebotomy and chelation improved glycemic control [20,21,22]. Reducing iron stores through frequent blood donation in healthy volunteers has also increased insulin sensitivity [23].

It is known that the hormone hepcidin is responsible for the regulation of iron metabolism. In addition to iron excess, hepcidin expression is influenced by inflammation. Hyperglycemia occurring in diabetes stimulates the synthesis of pro-inflammatory cytokines, thus leading to the development of chronic inflammation. Clinical trials have shown higher hepcidin levels in diabetic patients, which has correlated with concentration levels of Il-6 and ferritin [24,25]. Pro-inflammatory cytokines increase the expression of hepcidin, which stops the transport of iron from enterocytes to the blood and inhibits its release from macrophages, causing a decrease in its concentration in plasma and its accumulation in cells. The accumulation of iron in adipocytes results in reduced synthesis of adiponectin, thus increasing insulin resistance [26].

Diabetes mellitus is currently a global epidemic. The estimated number of people with diabetes worldwide is 171 million and it will reach 334 million in 2023 [27]. The main chronic complication of diabetes is diabetic kidney disease (DKD). DKD is an important and growing epidemiological and clinical problem. It is the most common cause of chronic kidney disease (CKD) and end-stage renal disease (ESRD) [28,29,30].

Anemia is one of the most commonly observed complications of chronic kidney disease CKD. The risk of anemia increases with the deterioration of renal function from approximately 27% in CKD stage 1–76% in stage 5 [31,32,33]. Moreover, 90% of dialysis patients had anemia [34,35]. DKD patients with anemia have a very high risk of cardiovascular diseases (CVD) and a much higher risk of death compared to people with DKD without anemia [36,37,38]. Anemia in DKD patients develops early, even before GFR is significantly lowered, and is more severe than in patients with non-diabetic kidney disease [39,40].

The pathogenesis of anemia in CKD is a multifactorial process. The most common causes include a decrease in erythropoietin (EPO) synthesis, iron deficiency (IDA), and chronic inflammation. In many patients with CKD, anemia is developed in the form of anemia of chronic disease, resulting from a chronic inflammatory process. The increased level of inflammatory cytokines leads to an increase in hepcidin expression, thus leading to the development of functional iron deficiency—a condition in which the body’s iron stores are normal or even increased, but the release of iron from them is not fast enough to ensure normal erythropoiesis. Serum saturation is low, and the concentration of ferritin is normal or high.

As in the published literature, data on iron parameters are scarce; therefore, the aim of the study was to assess novel iron parameters in patients with type 2 diabetes mellitus in relation to kidney function.

## 2. Materials and Methods

The study group consisted of 80 patients. All patients were hospitalized in the Teaching Hospital of Invasive Cardiology at the Medical University of Bialystok in Poland. The median age of patients in the study group was 70 years (min 42; max 88). There were 58 men (72.5%) and 22 women (27.5%) among the study group. All patients in the study group suffered from type 2 diabetes mellitus. Diabetes was defined by the current guidelines [41] or by the use of insulin or oral hypoglycemic agents. The control group consisted of 23 healthy volunteers (16 females) to obtained normal ranges for biomarkers.

The patients gave written informed consent for this study during hospitalization after receiving comprehensive information and learning about the purpose of the study. Data regarding the course of type 2 diabetes, chronic kidney disease, other chronic diseases, and risk factors were obtained from medical records. Diabetes mellitus in these patients was treated with biguanides, sulfonylurea derivatives, incretin drugs, sodium-glucose cotransporter (SGLT-2) inhibitors, and insulin. The treatment was conducted both in monotherapy as well as in combination therapy consisting of drug groups selected individually to the patient. Serum creatinine concentration was determined in all patients in the study group. Chronic kidney disease recognition was made according to KDIGO 2012 Clinical Practice Guideline for the Evaluation and Management of Chronic Kidney Disease [42]. CKD is defined as the presence of kidney damage or an estimated glomerular filtration rate (eGFR) of less than 60 mL/min/1.73 m^2^, persisting for three months or more. The CKD-EPI (Chronic Kidney Disease Epidemiology Collaboration) formula was also used to estimate the glomerular filtration rate [43] in the study group. The control group consisted of 23 healthy volunteers without type 2 diabetes and chronic kidney disease or other chronic diseases. Basic laboratory results (complete blood count, inflammatory parameters, electrolytes) and hepcidin, HIF1-alpha (Hypoxia-Inducible Factor 1-alpha), GDF-15 (Growth Differentiation Factor 15), sTfR (Soluble Transferrin Receptor), iron, and ferritin concentrations were performed in both the study and control groups. The study patients were divided according to the stage of CKD. eGFR < 60 mL/min/1.73 m^2^ was present in 21 patients, eGFR < 30 mL/min/1.73 m^2^ was present in 2 patients, and eGFR < 15 mL/min/1.73 m^2^ was observed in 1 patient. The patients were divided into two groups depending on the values of eGFR. Group 1 included patients with an eGFR < 60 mL/min/1.73 m^2^, and group 2 included patients with an eGFR ≥ 60 mL/min/1.73 m^2^.

The studied parameters were analyzed in relation to pharmacological treatment, the results of additional examinations, and coexisting diseases. Statistical analysis was performed using the Statistica 13.3 PL program. The aim of this study was to evaluate the relationship of specific factors affecting iron metabolism in diabetic patients (i.e., hepcidin, soluble transferrin receptor- sTfR, growth and differentiation factor 15-GDF-15, hypoxia-inducible factor 1 alpha- HIF-1 alpha with routinely determined iron markers, and kidney function). The approval of the Bioethics Committee of the Medical University of Bialystok was obtained (NO: R-I-002/7/2018).

### Statistical Analysis

We used the Shapiro–Wilk test to test for normality in the variable distributions. As all continuous variables were not normally distributed, we presented them as median and interquartile range values. Categorical variables were presented as the number of cases and percentages. The Mann–Whitney U tests and T-tests were used to determine the statistical significance of differences between variables. The correlations between variables were determined using multivariable Pearson’s correlations. Data are presented as rank correlations (r) with *p*-values. The threshold of statistical significance for all tests was set at *p* < 0.05. All analyses were performed using MS Excel (Microsoft, 2020, version 16.40, Redmond, WA, USA) and XL Stat (Addinsoft, 2020, version 2020.03.01, New York, NY, USA).

## 3. Results

Out of 80 patients, 24 patients were diagnosed with reduced renal function (their eGFR value was <60 mL/min/1.73 m^2^; 30%), whereas the remaining 56 patients (70%) showed normal glomerular filtration rates. Table 1 shows the characteristics of the studied and control populations. Anemia was found in 65% of the studied population according to the WHO definition (hemoglobin < 13 mg/dL for men and <12 mg/dL for women) [44].

The study group was found to have significantly higher hepcidin, sTfR, and GDF-15 levels, and lower hemoglobin and iron levels. When compared with patients with eGFR values ≥60 mL/min/1.73 m^2^ and <60 mL/min/1.73 m^2^, we found that patients with higher eGFR levels had higher hemoglobin, ferritin, and HIF-1 alpha levels; lower BNP; and were younger (Table 2).

In the studied group, there were statistically significant correlations between age and hemoglobin (*r* = −0.23, *p* < 0.05), eGFR (*r* = −0.36, *p* < 0.001), sTfR (*r* = −0.29, *p* < 0.01), and GDF-15 (*r* = −0.35, *p* < 0.001); between hemoglobin and eGFR (*r* = 0.37, *p* < 0.001), BNP (*r* = −0.39, *p* < 0.001), hepcidin-25 (*r* = 0.32, *p* < 0.01), and ferritin (r = 0.30, *p* < 0.01); between eGFR and BNP (*r* = −0.45, *p* < 0.001), sTfR (*r* = −0.31, *p* < 0.01), and GDF-15 (*r* = −0.24, *p* < 0.05); between hepcidin-25 and sTfR (*r* = −0.22, *p* < 0.05), GDF-15 (*r* =0 031, *p* < 0.01), and ferritin (*r* = 0.47, *p* < 0.001); and between sTfR and ferritin (*r* = −0.29, *p* < 0.05). All the correlations for the studied subgroups are presented in Table 3 (significant correlations are shown in bold). In the linear regression model, hepcidin was related to iron parameters (Table 4).

## 4. Discussion

In our study, we assessed—for the first time—several iron parameters, including HIF-1 alpha, in relation to routinely determined iron markers. However, we could not find statistically significant differences in iron parameters between patients with eGFR values ≥60 mL/min/1.73 m^2^ and <60 mL/min/1.73 m^2^, except for ferritin and HIF-1 alpha. For the study group, hepcidin was significantly related to iron parameters in the linear regression model.

In a recent cross-sectional study, Hayder and Kareem [45] described the possible role of resistin in the iron status pathway in patients with non-insulin-dependent diabetes mellitus and ESRD. In their study, they found that among 50 patients with type 2 diabetes mellitus and a mean eGFR level of 80 mL/min/1.73 m^2^, serum hepcidin and ferritin were higher when compared to the control group (where the mean eGFR was 89 mL/min/1.73 m^2^), while serum iron was significantly lower. The mean ages of the studied and control groups were not provided; it was only stated that volunteers were apparently normal, healthy subjects who more than 40 years old. We are fully aware that age- and sex-matching are difficult—in particular, when the studied group is almost 70 years old, as patients in the hospital typically are. No detailed clinical characteristics of the studied groups were provided. In many of our previous studies in patients with different types of renal replacement therapies, including kidney transplantation, we showed that the hepcidin was elevated in these patients [46,47,48]. As it was suggested, hepcidin-25 might be an important regulator of iron homeostasis under erythropoiesis in hemodialyzed patients [49]. In addition, elevated hepcidin-25 not only decreases the release of stored iron but also influences erythropoiesis [50]. Thus, alternatively, several studies indicated that hepcidin-25 was not a reliable marker for iron release monitoring and storage in dialyzed patients [51,52]. In our previous study, we reported higher hepcidin in the early stages of CKD [53]. However, in this study, we focused only on diabetic patients. Wagner et al. [54], in a cohort of 249 diabetic patients with CKD of any stage (with a mean age of 67 years and a mean eGFR level of 51 mL/min/1.73 m^2^), reported that elevated hepcidin was independently associated with ferritin and worsened kidney function (all *p* < 0.05).

Previously, we also reported that in patients ≥65 years, serum concentrations of GDF-15 were significantly higher in comparison with the younger group of patients with early stages of kidney disease [55]. GDF-15 is a member of the transforming growth factor-β cytokine superfamily and has been shown to be one of the regulators of iron status. GDF-15 expression is increased through oxidative stress and inflammation [56]. In the present study, we found that GDF-15 was higher in healthy volunteers but there was no difference between patients with eGFR values ≥60 mL/min/1.73 m^2^ and <60 mL/min/1.73 m^2^. In addition, in our previous study on 84 patients with CKD in the early stage (with a mean eGFR level of 67.7 ± 16 mL/min/1.73 m^2^, mean age of 69 years, and with 70% females), we found that sTfR was higher than in the control group [57]. sTfR is a dimeric protein present in serum, which is a part of the transferrin receptor after enzymatic cleavage [58]. In the study of Belo et al. [59], sTfR values were higher in hemodialyzed diabetic patients when compared to hemodialyzed non-diabetic patients and were significantly correlated with circulating iron, ferritin, transferrin saturation, hepcidin, MCH, and MCHC. There were no statistically significant differences in GDF-15 and hepcidin between diabetic and non-diabetic patients who were on hemodialysis in their study. Similar findings were observed in our study in diabetic patients who were not on dialysis.

sTfR reflects the total amount of transferrin receptors present in the body. The majority of the sTfR is found in the bone marrow. Assessing the level of the sTfR is useful to estimate the gross mass of erythroid bone marrow and may help to distinguish between the anemia of chronic diseases and iron deficiency anemia [60]. However, the lack of assay standardization and the wide variety in the cutoff values limits its interpretation in clinical practice [61].

In the published literature, we could not find a comprehensive study in iron metabolism diabetic patients within the early stages of CKD. We also assessed HIF-1 alpha in addition to other iron metabolism markers. HIF is the main transcription factor of hundreds of genes, including the EPO gene expressed in hypoxic tissues, and it adapts to a hypoxic environment [62,63]. HIF also controls the expression of the proteins playing a role in iron metabolism and utilization (the upregulation of transferrin, soluble transferrin receptor 1 (sTfr1), ceruloplasmin, divalent metal transporter 1 (DMT1), duodenal cytochrome b (Dcytb); and the downregulation of hepcidin). Increases in soluble transferrin receptor 1 improve iron availability, given its role as a carrier protein for transferrin required to import iron into the cell [64]. Researchers suggest that hepcidin suppression most likely results from the stimulation of erythropoiesis, as it seems that hepcidin is not a direct transcriptional target of HIF. Nowadays, hypoxia-inducible factor prolyl hydroxylase inhibitors (HIF-PHIs) represent a novel approach to the treatment of anemia in patients with CKD.

We found that levels of HIF-1 alpha are negligible in the studied population and were related to age only in patients with eGFR values ≥60 mL/min/1.73 m^2^. Whether the assessment of HIF-1 alpha would be a marker of efficient anemia therapy with HIF-PHIs is still a matter for further study.

Our study was cross-sectional, was completed using a single center, and included 80 patients that were subsequently admitted to the Department. We are aware that our population is relatively small, but for the assessment of several iron parameters, it appears to be sufficient to show the differences in relation to kidney function as well as to healthy controls. Our real-life data characterize everyday clinical practice with a substantial component of uncertainties and possible confounders.

## 5. Conclusions

In conclusion, a comprehensive assessment of iron status is rarely performed. Novel biomarkers of iron metabolism are not generally related to kidney function. Whether the assessment of HIF-1 alpha would be a marker of efficient anemia therapy with HIF-PHIs is still a matter for further study.

## Figures and Tables

**Table 1:** Characteristics of the studied and control populations.

	Study Participants (*N* = 80)	Control Cohort (*N* = 23)	*p* for the Comparison between Study Participants and the Control Cohort
Age, years; Median (IQR)	70 (11)	51 (8.6)	<0.001
eGFR by CKD-EPI, mL/min/1.73 m^2^; Mean (SD)	70.3 (20.8)	85.9 (15.3)	<0.001
Hemoglobin, g/dL; Mean (SD)	12.9 (1.3)	13.1 (1.4)	<0.001
Hepcidin, mg/mL; Median (IQR)	4.6 (5.3)	2.5 (1.2)	<0.001
sTfR, nmol/L; Mean (SD)	23.7 (7)	8.6 (2)	<0.001
GDF-15, pg/mL; Median (IQR)	1514 (1232)	584 (324)	<0.001
HIF-1 alpha, mg/mL; Median (IQR)	0 (0)	102.6 (112)	0.09
Fe, µg/dL; Mean (SD)	78.7 (24.9)	95.6 (24.7)	0.005
Ferritin, µg/L; Median (IQR)	99.6 (92.5)	104 (90.2)	0.57

**Table 2:** Comparisons between patients with and without chronic kidney disease.

	Study Participants (*N* = 80)	eGFR < 60 mL/min/1.72 m^2^ (*N* = 24)	eGFR > 60 mL/min/1.72 m^2^ (*N* = 56)	*p*
Age, years; Median (IQR)	70 (11)	73.5 (11.3)	69 (9.3)	0.03
Male; % (*N*)	73 (58)	75.0 (18)	71.4 (40)	0.75
BMI, kg/m^2^; Median (IQR)	30.6 (4.9)	33.3 (5.7)	30.4 (4.2)	0.08
Atrial fibrillation; % (*N*)	26 (21)	29 (7)	25 (14)	0.70
Hypertension; % (*N*)	91.3 (73)	92 (22)	91 (51)	0.93
Chronic heart failure; % (*N*)	34 (27)	33.3 (8)	34 (19)	0.96
Chronic coronary syndrome; % (*N*)	80 (64)	75 (18)	82.1 (46)	0.46
Acute coronary syndrome; % (*N*)	40 (32)	45.8 (11)	37.5 (21)	0.44
MAP, mmHg; Mean (SD)	94.3 (18.4)	96.4 (12.9)	93.4 (20.2)	0.49
Heart rate, beats per minute; Mean (SD)	73 (12.6)	71 (11.8)	70 (13)	0.98
Low-density lipoprotein cholesterol, mg/dL; Mean (SD)	86.4 (32.2)	88.1 (38)	82.2 (27.5)	0.46
High-density lipoprotein cholesterol, mg/dL; Mean (SD)	41.8 (11.4)	35 (15.5)	43.5 114)	0.04
TG, mg/dL; Mean (SD)	143 (56.6)	154.5 (53.5)	126 (61)	0.24
eGFR by CKD-EPI, mL/min/1.73 m^2^; Mean (SD)	70.3 (20.8)	50 (18)	81 (21.3)	<0.001
RBC 10^6^/mm^3^; Mean (SD)	4.3 (0.5)	4.2 (0.63)	4.3 (0.42)	0.09
Hemoglobin, g/dL; Mean (SD)	12.9 (1.3)	12.5 (1.5)	13.1 (1.3)	<0.001
BNP, pg/mL; Median (IQR)	246.3 (406.5)	454 (3877)	139 (186)	<0.001
Hepcidin, mg/mL; Median (IQR)	4.6 (5.3)	4.6 (4.9)	4.6 (5.4)	0.93
sTfR, nmol/L; Mean (SD)	23.7 (7)	26.3 (5.3)	23 (8.7)	0.08
GDF-15, pg/mL; Median (IQR)	1514 (1232)	1906 (1485)	1351 (1013)	0.1
HIF-1 alpha, mg/mL; Median (IQR)	0 (0)	0 (11.7)	0 (0)	0.02
Fe, µg/dL; Mean (SD)	78.7 (24.9)	75.5 (29.5)	78 (33.8)	0.28
Ferritin, µg/L; Median (IQR)	99.6 (92.5)	78 (49.6)	117 (108)	0.03
Diuretics medication prescribed at discharge; % (*N*)	77.5 (62)	83.3 (20)	75 (42)	0.36
Calcium channel blockers prescribed at discharge; % (*N*)	30 (24)	33.3 (8)	32 (18)	0.54
ACE medication prescribed at discharge; % (*N*)	84 (67)	88 (21)	82 (46)	0.47
BB medication prescribed at discharge; % (*N*)	88.8 (71)	96 (23)	86 (48)	0.09
Statin medication prescribed at discharge; % (*N*)	77.5 (62)	83 (20)	75 (42)	0.36

**Table 3:** Multivariable Pearson correlations between physical variables.

Hepcidin mg/mL	−0.03 (*p* = 0.9)	0.09 (*p* = 0.69)	0.34 (*p* = 0.1)	0.25 (*p* = 0.24)	−0.01 (*p* = 0.95)	0.3 (*p* = 0.16)	0.26 (*p* = 0.22)	−0.29 (*p* = 0.17)
0.04 (*p* = 0.74)	Age, years	−0.22 (*p* = 0.31)	−0.02 (*p* = 0.93)	0.19 (*p* = 0.37)	−0.03 (*p* = 0.9)	0.01 (*p* = 0.99)	0.12 (*p* = 0.58)	0.22 (*p* = 0.3)
−0.11 (*p* = 0.4)	**−0.32** (***p* = 0.01**)	eGFR, mL/min/1.73 m^2^	**0.44** (***p* = 0.03**)	−0.11 (*p* = 0.62)	−0.13 (*p* = 0.55)	−0.11 (*p* = 0.61)	−0.09 (*p* = 0.66)	−0.28 (*p* = 0.19)
**0.33** (***p* = 0.01**)	**−0.28** (***p* = 0.04**)	**0.35** (***p* = 0.01**)	HGB, g/dL	0.13 (*p* = 0.55)	0.01 (*p* = 0.99)	−0.12 (*p* = 0.59)	−0.16 (*p* = 0.46)	−0.15 (*p* = 0.47)
**0.47** (***p* < 0.001**)	−0.13 (*p* = 0.35)	0.05 (*p* = 0.72)	**0.31** (***p* = 0.02**)	Ferritin, µg/L	−0.08 (*p* = 0.69)	0.25 (*p* = 0.24)	0.11 (*p* = 0.06)	0.05 (*p* = 0.82)
−0.01 (*p* = 0.46)	0.01 (*p* = 0.99)	0.08 (*p* = 0.57)	0.17 (*p* = 0.21)	**0.27** (***p* = 0.04**)	Fe, µg/dl	0.09 (*p* = 0.66)	−0.07 (*p* = 0.72)	−0.25 (*p* = 0.23)
−0.05 (*p* = 0.72)	**−0.43** (***p* < 0.001**)	−0.02 (*p* = 0.91)	−0.02 (*p* = 0.9)	0.12 (*p* = 0.39)	0.05 (*p* = 0.7)	HIF-1 alpha pg, mg/mL	−0.16 (*p* = 0.45)	−0.11 (*p* = 0.61)
**0.28** (***p* = 0.04**)	**0.38** (***p* < 0.001**)	−0.18 (*p* = 0.18)	0.01 (*p* = 0.99)	0.09 (*p* = 0.53)	0.05 (*p* = 0.73)	−0.14 (*p* = 0.31)	GDF−15, pg/mL	0.07 (*p* = 0.74)
**−0.32** (***p* = 0.01**)	**0.33** (***p* = 0.01**)	−0.24 (*p* = 0.07)	−0.06 (*p* = 0.65)	**−0.29** (***p* = 0.03**)	−0.15 (*p* = 0.27)	−0.15 (*p* = 0.28)	0.12 (*p* = 0.38)	sTfR, nmol/L

Right side r value for group with eGFR < 60 mL/min/1.73 m^2^, left side for group with eGFR ≥ 60 mL/min/1.73 m^2^. Statistically significant correlations are in bold (*p* < 0.05).

**Table 4:** Results of linear regression analyses for serum hepcidin concentration.

Variables	β	(95% CI)		*p*
sTfR	−0.239	−0.431	−0.047	0.015
GDF-15	0.267	0.082	0.453	0.005
HIF-1 alpha	0.109	−0.074	0.292	0.239
Fe	−0.239	−0.416	−0.062	0.009
Ferritin	0.393	0.203	0.582	<0.0001
Hemoglobin	0.335	0.143	0.527	0.001
eGFR by CKD-EPI	−0.159	−0.368	0.050	0.134
Age	0.162	−0.039	0.362	0.113

## Data Availability

The data presented in this study are available on request from the corresponding author.
